# Phase-targeted erythropoietin derivatives for traumatic brain injury: bridging mechanisms to precision therapy

**DOI:** 10.3389/fneur.2025.1665405

**Published:** 2026-01-23

**Authors:** Yujin Sun, Bo Song, Yonglei Zhang, Yan Zhang, Lu Zhou

**Affiliations:** 1 Department of Emergency Medicine, Yantaishan Hospital, Yantai, China; 2 Department of Laboratory Medicine, Yantai Hospital of Traditional Chinese Medicine, Yantai, China

**Keywords:** traumatic brain injury, erythropoietin derivatives, neuroprotection, protein engineering, secondary injury phases

## Abstract

Traumatic brain injury (TBI) unfolds through a well-defined chronology—hyperacute excitotoxic and inflammasome bursts, acute apoptotic and blood–brain-barrier failure, and subacute neurovascular remodeling—that no single-pathway drug can adequately cover. Recombinant erythropoietin (EPO) limits secondary damage in animals, yet its erythropoietic drive and thrombotic liability have stalled clinical adoption. This review integrates structural biology, pharmacology and translational data on four engineered EPO derivatives—carbamylated EPO, asialo-EPO, darbepoetin alfa and the helix-B surface peptide (HBSP/cibinetide)—that decouple cytoprotection from red-cell stimulation. We first outline how specific modifications (carbamylation, desialylation, hyper-glycosylation or helix truncation) bias EPOR signaling toward PI3K–AKT and away from JAK2–STAT5. We then match each derivative to its optimal injury window. Meta-analyses of randomized trials suggest a possible trend toward lower short-term mortality without a consistent functional benefit or thrombotic signal. By integrating molecular mechanisms, experimental findings, and early clinical observations, this review outlines hypotheses and future trial frameworks for phase-targeted, erythropoietin-based neuroprotection. Further controlled studies are required to establish safety, efficacy, and optimal therapeutic timing before translation to routine clinical use.

## 1 Introduction

Traumatic brain injury (TBI) is a leading cause of death and long-term disability worldwide, particularly among young adults and the elderly. It results from external mechanical force leading to brain dysfunction, typically classified into mild, moderate, or severe based on clinical parameters such as the Glasgow Coma Scale. Globally, TBI affects an estimated 69 million individuals annually, imposing a significant socioeconomic burden due to its complex management and long-term neurological sequelae (1, 2).

The pathophysiology of TBI involves two major phases: the primary injury, characterized by the immediate mechanical damage to neurons, glial cells, and blood vessels; and the secondary injury, which develops over hours to days and includes excitotoxicity, oxidative stress, inflammation, mitochondrial dysfunction, and apoptosis (3). While advances in emergency care and surgical interventions have improved survival, effective pharmacological treatments to prevent or reverse secondary brain injury remain limited (4, 5). Current clinical management focuses primarily on supportive care and intracranial pressure control, without addressing the underlying cellular damage and neuroinflammatory cascades. Therefore, there is an urgent need to identify novel therapeutic agents that can modulate these secondary mechanisms and enhance neural repair (6, 7).

Erythropoietin (EPO) is a glycoprotein hormone traditionally recognized for its role in erythropoiesis, being mainly produced by the kidneys in response to hypoxia and acting through the erythropoietin receptor (EPOR) on erythroid progenitor cells (8). However, beyond its hematopoietic function, EPO has gained increasing attention for its tissue-protective effects in the central nervous system (9–11). EPO and EPOR are expressed in various brain regions, including neurons, astrocytes, and microglia, suggesting autocrine and paracrine neuroprotective roles (12, 13). Preclinical studies have demonstrated that EPO exerts neuroprotective effects through multiple mechanisms, such as anti-apoptotic signaling, anti-inflammatory modulation, blood–brain barrier stabilization, and promotion of neurogenesis. In various models of neurological injury—including stroke, spinal cord injury, and neonatal hypoxic–ischemic encephalopathy—EPO has shown beneficial effects on functional recovery and tissue preservation. In the context of TBI, experimental research has reported improved outcomes following EPO administration, including reduced lesion volume, enhanced cognitive recovery, and attenuated neuroinflammation (14–17).

Despite these promising findings, the translation of EPO into clinical treatment for TBI remains in its early stages. Challenges include optimizing the dosing regimen, minimizing hematological side effects, and understanding the therapeutic window for intervention. Furthermore, the precise mechanisms by which EPO confers neuroprotection in TBI are still being elucidated, particularly with regard to its effects on glial activation, synaptic function, and systemic responses to injury. Advances in protein engineering have since fostered a new generation of “EPO derivatives” designed to decouple tissue protection from erythropoiesis. Carbamylated EPO (CEPO) introduces homocitrulline modifications that abrogate EPOR-mediated red-cell stimulation yet retain anti-apoptotic and anti-inflammatory signaling; *in vitro* mechanical trauma and *in vivo* TBI models show CEPO neuroprotection comparable to rhEPO without hematological elevation (18). Removal of terminal sialic acids yields Asialo-EPO, a rapidly cleared molecule that nonetheless penetrates the CNS efficiently and confers robust protection in ischemia–reperfusion paradigms (19). Conversely, darbepoetin alfa, engineered with additional N-linked glycans, lengthens plasma half-life and has demonstrated dose- and time-dependent reduction of contusion volume, improved cerebrovascular reactivity, and antioxidant effects in experimental TBI (20). Collectively, these derivatives inaugurate an “EPO-based platform” that can be molecularly tuned along a spectrum of pharmacodynamics, offering unprecedented flexibility to match the pathophysiological timing and safety requirements of acute brain injury.

### 1.1 Objective of the review

Given the multifaceted pathophysiology of TBI and the emerging neuroprotective properties of erythropoietin, this review aims to systematically summarize the current understanding of EPO’s role in the treatment of traumatic brain injury. We begin by introducing the fundamental biological functions of EPO beyond erythropoiesis, followed by a detailed examination of the molecular and cellular mechanisms through which EPO may exert neuroprotection in TBI—such as regulation of glial cell survival, inflammation, cognitive function, and systemic biomarkers.

Furthermore, we review existing clinical evidence regarding the safety, dosing, and efficacy of EPO in TBI patients, and highlight challenges that have limited its clinical translation. Finally, we provide perspectives on future directions in this field, including opportunities for optimizing EPO-based therapies and exploring derivative compounds with reduced hematopoietic activity. By integrating basic, preclinical, and clinical findings, this review seeks to clarify the therapeutic potential of EPO in TBI and identify critical knowledge gaps for future research.

## 2 Methods of the review

### 2.1 Search strategy

We searched PubMed, Web of Science, and the Cochrane Library, and screened trial registries (ClinicalTrials.gov and WHO ICTRP) for ongoing/completed human studies and device/drug entries. We also hand-searched reference lists of eligible articles and relevant reviews and consulted regulatory webpages (FDA, EMA) for designations or approvals mentioned in the text. Boolean combinations (title/abstract and keywords) were used, tailored to each database. Core strings included: “erythropoietin,” “EPO,” “carbamylated erythropoietin,” “CEPO,” “asialo-EPO,” “darbepoetin,”“HBSP,”“ARA290,” “helix-B surface peptide,” “traumatic brain injury,” “TBI,”“neurotrauma,” “controlled cortical impact,” “fluid percussion,” plus delivery terms (such as “intranasal,” “hydrogel,” “microparticle,” “liposome,” “transferrin receptor,” “fusion protein”).

### 2.2 Eligibility criteria

#### 2.2.1 Inclusion

Peer-reviewed articles in English; (a) preclinical CNS injury models with explicit TBI relevance (controlled cortical impact (CCI), fluid percussion injury (FPI), closed-head injury) evaluating EPO or derivatives; (b) human interventional or observational studies in TBI or acute CNS injury reporting clinical outcomes, safety, PK/PD, or validated biomarkers; (c) delivery-strategy papers with CNS-directed EPO/derivatives; (d) systematic reviews/meta-analyses informing efficacy/safety.

#### 2.2.2 Exclusion

Non-peer-reviewed content; isolated case reports without mechanistic or dosing relevance; non-CNS contexts unless directly informing delivery, PK, or receptor/signaling controversies; articles lacking original data (except high-quality guidelines or regulatory notices).

#### 2.2.3 Study selection

Authors independently screened titles/abstracts, assessed full texts against criteria, and resolved disagreements by consensus. When multiple reports overlapped, the most comprehensive or recent dataset was prioritized. The literature search was completed on October, 2025.

#### 2.2.4 Data extraction

We abstracted model/clinical design, population/species, injury paradigm, derivative identity/dose/route/timing, comparators, outcomes (lesion volume, neurobehavior, cytokines; for human studies: mortality, functional scales, AEs, thrombotic events), and key mechanistic readouts.

### 2.3 Risk-of-bias and quality appraisal

Evidence was synthesized by (1) injury phase (hyperacute, acute, subacute), (2) derivative class (CEPO, asialo-EPO, darbepoetin, HBSP), and (3) endpoint tier (mechanistic → preclinical functional → human safety/biomarkers → human clinical outcomes). RCTs and meta-analyses carried the greatest weight for clinical claims; early-phase or surrogate-endpoint signals were explicitly labeled as hypothesis-generating. Where controversies exist (e.g., EPOR–βcR signaling), we collated supportive and opposing evidence and flagged knowledge gaps.

## 3 Structural basis and engineering strategies for EPO-derived neuroprotective agents

Erythropoietin (EPO) is a 165-amino acid glycoprotein belonging to the type I cytokine superfamily (21). Its biological effects are mediated by binding to the erythropoietin receptor (EPOR), a homodimeric transmembrane receptor expressed not only on erythroid progenitors but also on neurons, astrocytes, microglia, oligodendrocyte precursor cells (OPCs), and endothelial cells in the central nervous system (CNS). The canonical EPO–EPOR interaction triggers downstream signaling via JAK2/STAT5, PI3K/AKT, and MAPK/ERK pathways, orchestrating cell survival, proliferation, anti-apoptotic responses, and inflammation resolution (22–24).

### 3.1 EPO-EPOR interaction: structural insights

The functional domains of EPO that mediate its receptor binding and biological activity have been precisely mapped. EPO contains two high-affinity receptor binding sites, termed Site 1 and Site 2. Site 1 binds to the first EPOR molecule with high affinity, initiating receptor dimerization, while Site 2 engages the second EPOR molecule with lower affinity, stabilizing the complex and triggering signal transduction. This asymmetric binding is essential for full receptor activation and downstream signaling (25, 26).

Key residues such as Arg-103, Ser-100, and Leu-108 are crucial for receptor recognition. Structural studies have shown that selective mutations or chemical modifications of these residues can alter receptor affinity and signaling bias—opening avenues to decouple neuroprotective signaling from erythropoiesis (27, 28).

### 3.2 Rational engineering: decoupling neuroprotection from erythropoiesis

The therapeutic limitation of native EPO stems from its hematopoietic potency, which increases red blood cell mass, hemoglobin concentration, and ultimately thromboembolic risk—particularly problematic in TBI or stroke patients (29, 30). Therefore, engineering strategies have focused on structural modifications that preserve neuroprotective signaling while eliminating or minimizing erythropoietic effects.

Three main strategies have emerged:

*Chemical modification*: Carbamylation of lysine residues generates carbamylated EPO (CEPO), which lacks affinity for the classical EPOR homodimer but retains tissue-protective function via alternative receptor complexes, possibly EPOR–β common receptor (βcR) heterodimers (31). CEPO has shown equivalent efficacy to rhEPO in preclinical TBI and stroke models, with a markedly reduced hematologic profile (32, 33).

*Glycosylation manipulation*: Native EPO contains three N-linked and one O-linked glycosylation sites that modulate serum half-life and receptor binding. Removal of terminal sialic acids results in asialo-EPO, which is rapidly cleared from circulation but exhibits enhanced CNS penetration and potent neuroprotection (34). In contrast, darbepoetin alfa incorporates additional N-linked oligosaccharides, increasing molecular weight and prolonging plasma half-life, thereby supporting sustained neuroprotective exposure with fewer injections (35).

*Peptide truncation and mimetics*: Efforts to minimize molecule size while retaining function have led to the development of helix-B surface peptide (HBSP) and EPObis, short synthetic peptides derived from the EPO binding interface (36, 37). These molecules do not bind classical EPOR but activate downstream tissue-protective signaling cascades, likely via noncanonical receptors. Their low immunogenicity and synthetic accessibility make them attractive candidates for future development.

Additionally, site-directed mutagenesis has yielded point-mutated EPO variants (e.g., EPO-S104I or EPO-R103K) that exhibit selective activation of cytoprotective over erythropoietic pathways (38). These approaches underscore the principle of “biased agonism,” where specific conformational receptor states are engaged to yield favorable therapeutic effects with minimal side consequences.

### 3.3 Non-protein engineering approaches: small molecules and conjugates

Protein truncation is not the only route to decouple cytoprotection from erythropoiesis. A parallel research stream is generating small peptides, antibody–EPO fusions and nano-conjugates that engage EPOR-dependent tissue-protective pathways while further improving brain penetrance, pharmacokinetics and manufacturability.

#### 3.3.1 Peptidomimetic agonists

The 11-residue pyro-glutamate helix-B surface peptide (pHBSP) reproduces the amphipathic face of EPO’s helix B yet cannot dimerise the classical EPOR. In a rat model of mild TBI complicated by hemorrhagic shock, pHBSP given up to 110 min post-insult halved cortical contusion volume and normalized neurological scores without affecting hematocrit (39). A separate closed-head-injury study confirmed improved Morris-water-maze performance and reduced microglial activation after intraperitoneal pHBSP pulses (40). The 19-mer JM4 peptide provides similar protection: a single dose 30 min after controlled-cortical-impact reduced TUNEL-positive neurons by ~50% and improved composite neuroscores at 24 h (41). More recently, the clinical-grade dipeptidyl variant ARA290 (cibinetide) prevented infarct expansion via β-common-receptor signaling in a mouse focal-ischemia model without stimulating erythropoiesis evidence that ultra-short EPOR agonists can deliver brain protection across CNS (42).

#### 3.3.2 Receptor-targeted fusion constructs

Low native BBB permeability forces high systemic EPO doses, raising thromboembolic risk. One workaround is to fuse EPO to a monoclonal antibody directed at the transferrin receptor (cTfRMAb-EPO). This bispecific construct exploits receptor-mediated transcytosis to raise cerebrospinal concentrations >5-fold and afforded a 40% reduction in ischemic lesion volume at one-tenth the rhEPO dose in murine middle-cerebral-artery occlusion (43). Although tested in stroke rather than TBI, the study demonstrates a generalizable strategy for brain-directed cytokine delivery.

#### 3.3.3 Nano- and liposomal formulations

Poly-(lactic-co-glycolic acid) nano-erythropoietin (PLGA-EPO-NP) cut cortical infarct size 10-fold more effectively than free rhEPO and achieved functional rescue at 1/10th the dose in neonatal hypoxia–ischemia (44). A cholic-acid-coated PLGA system loaded with EPO further improved neurological recovery in adult rat stroke while enhancing BBB penetration and plasma half-life (45). Liposomal encapsulation has also been applied to asialo-EPO (AEPO); in rat ischemia–reperfusion, a single AEPO-liposome injection reduced TTC -defined infarcts and improved motor scores more robustly than free AEPO (34). Recent advances in microparticle delivery, such as the biodegradable PLGA microparticle system reported by DeJulius (46), have enabled sustained intraparenchymal EPO release and improved blood–brain-barrier penetration, representing an important translational step toward precise CNS dosing.

### 3.4 Controversies and knowledge gaps in EPO receptor biology

Despite extensive evidence that engineered EPO derivatives (e.g., CEPO, HBSP/ARA290) can elicit cytoprotection without stimulating erythropoiesis, the precise receptor mechanisms underlying these effects remain controversial (47, 48). The prevailing model proposes that tissue-protective signaling requires the formation of a heteromeric complex between the erythropoietin receptor (EPOR) and the β-common receptor (βcR, CD131), which activates anti-apoptotic and anti-inflammatory cascades independent of hematopoietic STAT5 signaling (49). Several studies have failed to confirm robust expression or functional signaling of EPOR (and by implication accessory receptors such as βcR) in non-hematopoietic human cells including endothelial, cardiac, renal, and some neural types (48). Additionally, recent structure–function analyses of EPO analogs indicate that some tissue-protective effects might arise from partial agonism at classical EPOR or from interactions with yet unidentified accessory molecules (50, 51). Together, these findings highlight a fundamental knowledge gap in how EPO and its derivatives engage receptor systems in the brain and spinal cord, emphasizing the need for high-resolution structural and *in vivo* functional studies to delineate receptor composition and downstream bias.

## 4 Key EPO derivatives—structural features, pharmacology, and neuroprotective profiles

**Figure 1:**
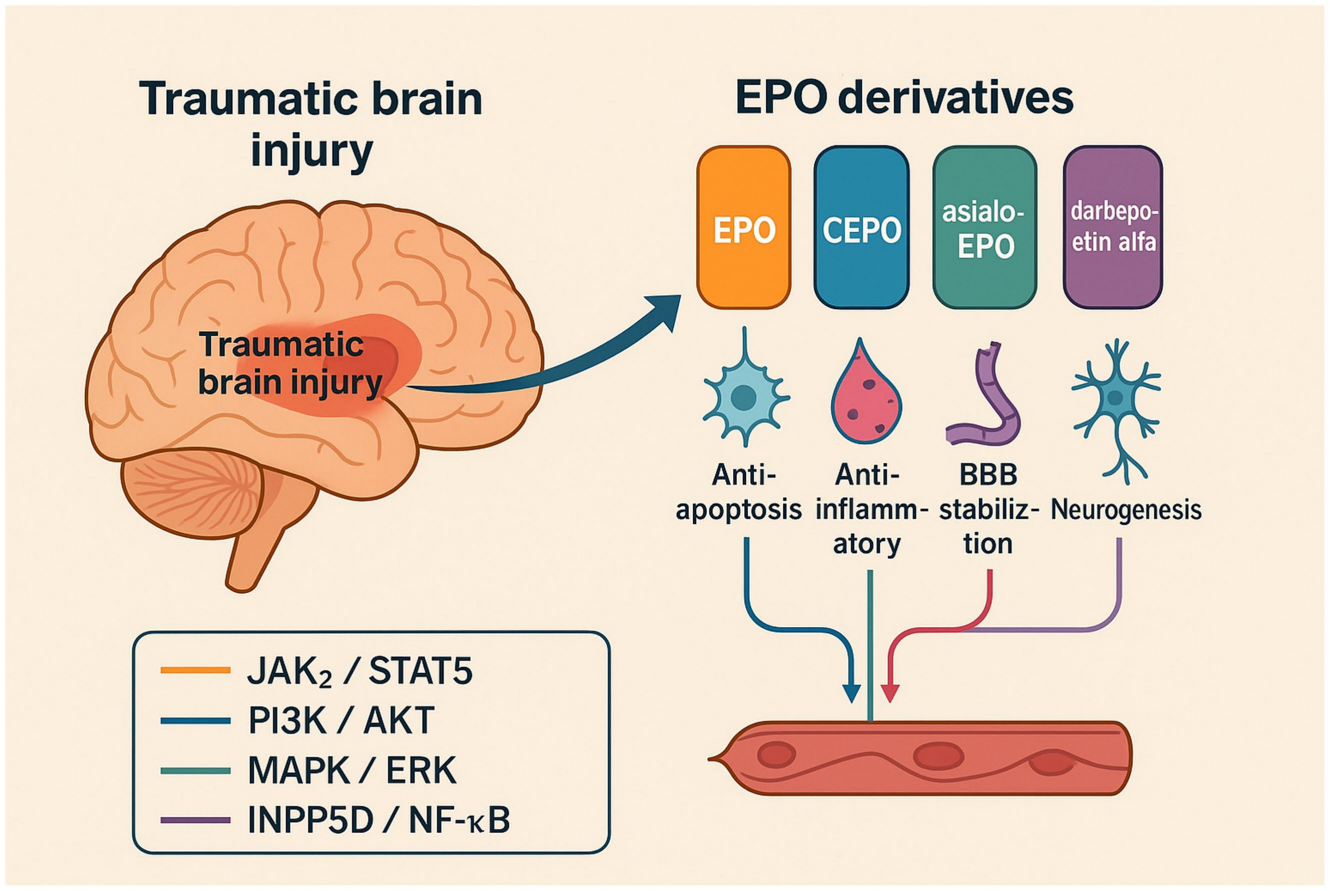
Schematic overview of erythropoietin (EPO) and engineered derivatives in traumatic brain injury (TBI). The diagram illustrates phase-specific mechanisms by which EPO and its engineered derivatives (carbamylated EPO, asialo-EPO, darbepoetin alfa, and HBSP/cibinetide) mitigate secondary brain injury. HBSP and CEPO are βcR-biased ligands that preferentially activate cytoprotective PI3K–AKT and NF-κB pathways, whereas darbepoetin alfa retains STAT5 signaling associated with erythropoiesis. Color coding: orange = JAK2/STAT5; blue = PI3K/AKT; teal = MAPK/ERK; purple = INPP5D/NF-κB.

Protein-engineering efforts have yielded several “next-generation” EPO molecules that preserve tissue-protective signaling while attenuating, or eliminating, classical erythropoiesis. Below we highlight the four best-studied representatives—carbamylated EPO (CEPO), asialo-EPO, darbepoetin alfa, and the helix-B surface peptide ARA290 (HBSP)—and discuss how their structural changes translate into distinct pharmacokinetic and safety profiles, alongside evidence for neuroprotection in TBI and related CNS injuries (Table 1 and Figure 1).

**Table 1:** Comparative snapshot of lead EPO derivative.

Molecule	Key structural modification	Serum half-life (vs. rhEPO)	Hematopoietic activity	Major neuroprotective evidence (TBI/CNS)	Safety notes	Route of administration and dose (μg/kg)
CEPO ()	Carbamylation of Lys residues → homocitrulline	≈4–6 h (similar)	None	↓ lesion size; ↑ neurogenesis in rat CCI and FPI models (32)	No Hb/Hct rise; good systemic tolerance	i.p./i.v. (50–1,000)
Asialo-EPO	Enzymatic desialylation of N-/O-glycans	<2 min (very short)	None	Robust stroke protection; emerging TBI data (59)	Requires frequent dosing; minimal systemic effects	i.v./i.n. (100–500)
Darbepoetin alfa	Two extra N-glycans (+7 kDa)	3 × longer (20–25 h)	High	Dose- & time-dependent contusion reduction in rat TBI; improved CBF (52)	Hb/Hct elevation; thrombotic vigilance needed	i.v./s.c. (10–50)
HBSP/ARA290	11-aa helix-B mimetic peptide (pyroGlu-HB)	<10 min	None	Comparable to rhEPO in mTBI + hemorrhage; βcR-mediated protection (42)	Early human PK/safety only; short exposure	i.v./i.n./hydrogel (10–100)

rhEPO reference half-life ≈ 5–8 h (i.v.)/24 h (s.c.). CCI, controlled cortical impact; FPI, fluid percussion injury; i.v., intravenous; i.p., intraperitoneal; i.n., intranasal; s.c., subcutaneous.

### 4.1 Carbamylated EPO

Selective carbamylation of lysine residues converts them to homocitrulline, abolishing high-affinity binding to the classical EPOR homodimer yet permitting engagement of an EPOR–β-common-receptor (βcR) heterocomplex. In rodent controlled-cortical-impact and fluid-percussion models, CEPO (10–50 μg/kg) reduced lesion volume, preserved hippocampal neurons, and improved sensorimotor scores without increasing hematocrit. Its plasma half-life is similar to native rhEPO [≈4–6 h, intravenous therapy (i.v.)], but the absence of erythropoietic drive markedly improves the therapeutic window (32).

### 4.2 Asialo-EPO

Enzymatic removal of terminal sialic acids shortens serum persistence to <2 min yet facilitates rapid CNS transit and virtually abolishes red-cell stimulation. *In vivo*, asialo-EPO protects against cerebral ischemia-reperfusion, attenuating infarct size and neuroinflammation; comparable experiments in closed-head-injury models are ongoing. Repeated dosing is required, but systemic adverse effects remain negligible (52).

### 4.3 Darbepoetin alfa

Introducing two extra N-linked glycan chains (five vs. three in rhEPO) increases molecular weight (≈37 kDa) and extends plasma half-life by ~3-fold (≈20–25 h i.v.). Despite retaining erythropoietic potency, darbepoetin (25–50 μg/kg) administered within 6 h post-impact injury in rats cut contusion volume by >70% and improved cerebral blood-flow reactivity. Vascular stabilization appears central to its neuroprotective effect, but hematological monitoring remains mandatory (52–54).

### 4.4 Helix-B surface peptide/ARA290

HBSP is an 11-mer mimetic of the hydrophilic helix-B domain of EPO; its N-terminal pyroglutamate variant (ARA290) displays high affinity for EPOR–βcR, negligible erythropoiesis, and a very short systemic half-life (<10 min). In mice subjected to mild TBI plus hemorrhagic shock, ARA290 (30 μg/kg, b.i.d.) matched rhEPO in cortical preservation and cognitive recovery, implicating anti-inflammatory βcR signaling as the dominant mechanism. Early-phase clinical testing in neuropathic pain has reported an excellent safety profile (42).

In addition to CEPO, asialo-EPO, darbepoetin alfa and HBSP, other engineered or gene-delivered EPO constructs have also demonstrated potent neuroprotective efficacy across diverse CNS contexts. For example, systemic gene delivery of EPO via recombinant adeno-associated virus vectors protected photoreceptors in the retinal degeneration slow (rds) mouse model, preserving retinal structure and visual function. In a subsequent study, rationally designed AAV2 and AAVrh8R capsids enabled improved transduction efficiency in both retina and brain, achieving sustained EPO expression that conferred long-term neuronal survival and reduced gliosis after neurotrauma (53, 54). Similarly, transgenic and plasmid-based neuronal overexpression models further confirmed that EPO-mediated activation of PI3K–AKT and STAT5 signaling attenuates apoptosis and inflammation in multiple injury paradigms (55, 56).

These derivatives collectively demonstrate that structure-guided modulation of EPOR signaling can finetune the balance between neuroprotection and hematological safety. CEPO and HBSP exemplify the “non-erythropoietic” class suited for acute therapy, whereas darbepoetin offers prolonged exposure at the cost of closer monitoring. Asialo-EPO highlights how ultra-rapid clearance can still yield robust CNS benefit, provided penetration is efficient and dosing is optimized. Together, they constitute a versatile toolkit for tailored intervention across the temporal phases of TBI and other CNS injuries.

## 5 Comparative neuroprotective mechanisms of EPO derivatives

Although all EPO-based agents hinge on EPOR-dependent signaling, their structural tweaks bias downstream pathways in subtly different ways. Here we compare the four “lead” derivatives—CEPO, asialo-EPO, darbepoetin alfa, and HBSP (ARA290)—across the principal mechanistic axes that govern secondary brain injury.

### 5.1 Anti-apoptotic and pro-survival signaling

Native rhEPO activates JAK2/STAT5, PI3K/AKT, and MAPK/ERK to up-regulate Bcl-2, inhibit caspase-3, and preserve mitochondrial integrity (57). CEPO and darbepoetin retain this full kinase triad; both reduced cortical caspase-3 activity and TUNEL-positive neurons in rat controlled-cortical-impact (CCI) models, with CEPO showing equal efficacy to rhEPO at 30 μg/kg but without hematocrit elevation (58). Asialo-EPO and HBSP, by contrast, favor PI3K/AKT over STAT5—enough to block apoptosis yet insufficient to drive erythropoiesis; their short systemic exposure nonetheless yields robust neuroprotection when dosed repeatedly within the first 24 h after injury (59).

### 5.2 Modulation of neuro-inflammation

Microglial and peripheral innate-immune activation amplify neuronal loss after TBI (60). All four derivatives suppress NF-κB and NLRP3 inflammasome activity, but potency diverges: HBSP shows the strongest bias toward anti-inflammatory β-common-receptor (βcR) signaling, shifting microglia to an M2 phenotype and lowering IL-1β, TNF-α and IL-6 levels in mouse mild-TBI + hemorrhagic-shock models (61). CEPO similarly dampens pro-inflammatory cytokines yet maintains modest STAT5 activation, whereas darbepoetin’s longer half-life prolongs cytokine suppression but at the expense of closer thrombotic surveillance. Asialo-EPO exerts rapid, transient NF-κB inhibition, suiting early-phase intervention but necessitating repeated pulses for sustained benefit (34).

### 5.3 Vascular protection and BBB stabilization

Secondary hypoperfusion and BBB leakage drive oedema and oxidative stress. Darbepoetin, with its extended residence time, excels at endothelial protection—improving cerebrovascular reactivity, reducing Evans-blue extravasation, and preserving tight-junction proteins in rat CCI within a 6-h therapeutic window (43, 62). CEPO and HBSP also safeguard the microvasculature but clear more rapidly, suggesting a role as acute “bolt-on” agents or as part of multimodal regimens. Asialo-EPO lacks durable vascular effects; its strength lies in quickly crossing a compromised BBB to reach parenchymal targets.

### 5.4 Promotion of neurogenesis and synaptic plasticity

All derivatives enhance hippocampal progenitor proliferation and dendritic spine density, yet magnitude tracks with EPOR engagement duration. Darbepoetin induces sustained dentate-gyrus neurogenesis for up to 14 days post-TBI, while CEPO achieves a comparable boost over 72 h without hematological cost. HBSP’s ultra-short half-life limits neurogenic effects unless delivered in controlled-release matrices, an area now explored with PEGylated hydrogels (62). Asialo-EPO improves long-term potentiation (LTP) but requires multiple administrations, aligning with its pharmacokinetic profile (43).

Collectively, the mechanistic comparison underscores a “plug-and-play” paradigm: derivative choice can be matched to the evolving biology of TBI, maximizing efficacy while minimizing risk. CEPO and HBSP shine where anti-inflammatory speed matters; darbepoetin suits prolonged vascular and regenerative support; asialo-EPO offers rapid parenchymal access with minimal systemic exposure (Table 2).

**Table 2:** Timing, dosing, and derivative-specific nuances.

Phase after injury	Dominant pathophysiology	Recommended derivative	Rationale	Delivery route
0–6 h (hyperacute)	Excitotoxicity, oxidative burst	Asialo-EPO or HBSP	Fast BBB penetration and strong anti-oxidant/anti-inflammatory bias	i.v., i.n.
6–48 h (acute)	Apoptosis, microglial activation, BBB breakdown	CEPO	Balanced anti-apoptotic + anti-inflammatory signaling without raising Hb/Hct	i.v., i.p.
>48 h–2 weeks (subacute)	Neurovascular remodeling, neurogenesis	Darbepoetin α (careful hematology)	Prolonged endothelial and neurogenic support	s.c., depot gel

i.v., intravenous; i.p., intraperitoneal; i.n., intranasal; s.c., subcutaneous; depot, sustained-release depot formulation.

## 6 From bench to bedside: evidence, pharmacokinetics, and safety

### 6.1 Pharmacokinetic profiles and systemic safety

The engineered molecules span a remarkable range of exposure kinetics. Asialo-EPO and HBSP clear from plasma in less than 10 min but achieve brain-to-plasma ratios approaching unity almost immediately—an ideal fit for the hyperacute inflammatory burst. CEPO mirrors the six-hour half-life of rhEPO, allowing twice-daily coverage through the apoptotic phase, while darbepoetin delivers day-long endothelial support with a single infusion. Safety tracks with these kinetics. In rodent and large-animal studies, CEPO, asialo-EPO and HBSP have proven hematologically inert (63, 64). By contrast, darbepoetin and rhEPO predictably raise hemoglobin (65); nevertheless, multi- network meta-analysis encompassing nearly 2,000 TBI patients detected no signal for deep-vein thrombosis or pulmonary embolism, suggesting that with judicious monitoring the thrombotic risk remains manageable (66, 67).

### 6.2 Clinical translation status

Human evidence is still dominated by native rhEPO, yet even these data hint at clinical utility. These studies form the current clinical evidence base for erythropoietin in TBI. The 606-patient EPO-TBI randomized trial failed to improve global neurological outcome at 6 months but cut in-hospital mortality from 16 to 11%, a benefit concentrated in transfusion-naïve patients. Aggregated analyses of erythropoietin (EPO) in traumatic brain injury—including 13 randomized controlled trials encompassing >3,000 patients—have reported modest but variable reductions in short-term mortality (relative risk ≈ 0.70–0.85, 95% CI 0.50–1.00) without consistent improvement in long-term functional outcomes, indicating no statistically significant increase in DVT or PE risk (66, 67). Hematology monitoring for hemoglobin and hematocrit is recommended in translational or clinical contexts. At present, robust human evidence for engineered EPO derivatives remains sparse. Darbepoetin alfa has been evaluated primarily in small, early-phase safety or PK studies without conclusive neurological endpoints, while CEPO-Fc and asialo-EPO are undergoing initial human testing or remain in preclinical development. Accordingly, extrapolation from rhEPO trials to these analogs should be considered hypothesis-generating rather than confirmatory.

### 6.3 Stage-matched derivative selection

Secondary injury evolves in waves: an explosive burst of excitotoxicity and inflammasome activation within the first few hours, followed by apoptosis, BBB failure and microvascular dysregulation over days, then a protracted phase of synaptic re-wiring and neurogenesis. Each derivative maps naturally onto one or more of these windows.

#### 6.3.1 Hyperacute (<6 h)

Agents that reach the parenchyma quickly and bias signaling toward β-common-receptor–dependent anti-inflammatory pathways are ideal. HBSP (ARA290) and asialo-EPO, both devoid of erythropoietic drive and cleared in minutes, suppress NF-κB and NLRP3 activity almost immediately after systemic injection, curbing microglial toxicity before cell-death cascades gain momentum (68).

#### 6.3.2 Acute (6–48 h)

Apoptotic death and BBB leakage dominate. CEPO, with a modest six-hour half-life and balanced AKT/STAT5 activation, can be given once or twice daily to blanket this interval; animal data show lesion reductions and cognitive rescue without hematocrit elevation (69).

#### 6.3.3 Subacute (>48 h)

Vascular remodeling and neurogenesis require days of signaling. Long-acting darbepoetin alfa extends plasma exposure to ~24 h, augments endothelial nitric-oxide synthase, and restores cerebrovascular reactivity in rodent CCI; its neurogenic effects persist for 2 weeks (70, 71). Close hematological surveillance is essential, but where anemia or vasospasm threaten outcome, the benefit–risk ratio may be favorable.

A staged protocol that begins with HBSP or asialo-EPO in the emergency department, transitions to CEPO infusions in the neuro-ICU, and finishes with a limited course of darbepoetin during rehabilitation is conceptually attractive (72). Small adaptive trials could test such sequencing while minimizing exposure to any single agent.

## 7 Future directions—precision deployment and translational priorities

Success for EPO-based therapy now rests not on discovering an all-purpose super-agonist but on matching each derivative—and its delivery route—to the evolving biology of injury and the bedside realities of critical care. Hyperacute pathophysiology is dominated by inflammasome activation and mitochondrial collapse; peptides such as HBSP or ultrashort asialo-EPO reach parenchyma within minutes, bias signaling toward anti-inflammatory β-common-receptor pathways and extinguish that first toxic wave. As apoptosis, blood–brain-barrier failure and endothelial dysfunction emerge over the ensuing hours, CEPO offers balanced anti-apoptotic and anti-inflammatory cover without raising hematocrit (73). Days later, when neurovascular remodeling and neurogenesis dictate long-term outcome, the twenty-hour half-life of darbepoetin sustains endothelial nitric-oxide synthesis and hippocampal progenitor recruitment (74). The logical clinical protocol therefore unfolds in phases: a rapid-acting peptide in the emergency department, CEPO infusions in the neuro-ICU, and one or two darbepoetin doses during early rehabilitation—an architecture already being modeled in small adaptive trials.

Logistics are critical. Intranasal CEPO-Fc, now in phase I safety testing, exploits olfactory pathways to achieve high brain-to-plasma ratios with negligible systemic exposure (75, 76), while injectable four-arm PEG-hydrogels that secrete HBSP for a week can be placed into peri-contusional tissue during decompressive craniectomy (77). Even cell therapy is moving into the frame: mesenchymal stromal cells pre-conditioned with CEPO double graft survival and synaptic rescue in chronic-TBI rats and enter human study later this year (78). Combination pharmacology—CEPO plus minocycline for broader cytokine control, darbepoetin with cilostazol for synergistic vascular stabilization—is being folded into REMAP-CAP-style platform trials that can adapt doses and arms as Bayesian probabilities of benefit evolve (79).

Parallel advances in diagnostics will enable biomarker-guided derivative choice. Early surges in serum IL-6 or S100B identify inflammasome-high phenotypes likely to respond to HBSP (80); persistent cerebral lactate or delayed spikes in VEGF flag microvascular fragility that may favor darbepoetin (81). High-throughput phospho-proteomics and AI-guided docking are now delivering small-molecule EPOR ligands that activate AKT without touching STAT5, the first of which have just cleared pre-clinical toxicology (82).

Several early-phase exploratory studies are currently evaluating engineered erythropoietin (EPO) derivatives or modified delivery strategies in acute central nervous system (CNS) injury. These include pharmacokinetic, safety, and formulation-optimization investigations designed to translate receptor-selective or depot-release strategies from preclinical models into clinical testing. While such work reflects growing translational interest, these studies remain preliminary and should be interpreted as proof-of-concept efforts rather than confirmatory clinical trials. Overall, these derivatives are best regarded as investigational agents under early development for neuroprotective or anti-inflammatory applications, rather than approved or officially designated therapeutics. Information on ongoing or completed clinical studies was retrieved from ClinicalTrials.gov and the WHO ICTRP (search completed in October 2025).

## 8 Conclusion and outlook

Traumatic brain injury represents a uniquely complex therapeutic target: its pathobiology evolves in distinct phases, no single pathway dominates, and any intervention must contend with the practical constraints of neuro-critical care. The cumulative evidence reviewed here shows that erythropoietin and, more convincingly, its next-generation derivatives have moved beyond proof-of-concept. Structure-guided engineering—carbamylation, glyco-remodeling, peptide miniaturization—has created a modular “EPO-based platform” that can be dialed toward rapid anti-inflammatory action (asialo-EPO, HBSP), balanced cytoprotection without erythropoiesis (CEPO), or prolonged vascular and neurogenic support (darbepoetin alfa). Pre-clinical data across TBI, stroke and spinal-cord injury confirm that these molecules mitigate apoptosis, blunt inflammasome activity, stabilize the blood–brain barrier and foster neuroregeneration, all while minimizing hematological risk.

Early clinical signals are encouraging—most notably a reproducible mortality benefit with native rhEPO and favorable safety read-outs for darbepoetin and CEPO-Fc—but functional gains remain elusive, largely because trials have not yet aligned derivative selection, dosing window and delivery route with the temporal biology of injury. That alignment is now within reach. Intranasal fusion proteins, hydrogel depots and cell-priming strategies can deliver phase-specific exposure; adaptive platform trials can test staged or combination regimens efficiently; and emerging biomarkers (IL-6, S100B, VEGF, miR-451) promise real-time guidance of derivative choice. Regulatory momentum is building, with fast-track and orphan designations signaling a clear path to licensure.

The next decade should therefore pivot from molecular innovation to translational execution. Success will hinge on integrating precision delivery technologies, biomarker-guided algorithms and adaptive trial designs to match each patient’s evolving pathophysiology with the optimal EPO derivative. If that coordination is achieved, EPO-based therapeutics have the potential not merely to reduce mortality but to transform long-term neurological recovery after TBI and, by extension, other acute CNS insults.
